# Diffusion-Based Design of Multi-Layered Ophthalmic Lenses for Controlled Drug Release

**DOI:** 10.1371/journal.pone.0167728

**Published:** 2016-12-09

**Authors:** Andreia F. R. Pimenta, Ana Paula Serro, Patrizia Paradiso, Benilde Saramago, Rogério Colaço

**Affiliations:** 1 Department of Mechanical Engineering and IDMEC, Instituto Superior Técnico, Universidade de Lisboa, Lisbon, Portugal; 2 Centro de Química Estrutural, Instituto Superior Técnico, Universidade de Lisboa, Lisbon, Portugal; 3 CIIEM, Instituto Superior de Ciências da Saúde Egas Moniz, Caparica, Portugal; Helsingin Yliopisto, FINLAND

## Abstract

The study of ocular drug delivery systems has been one of the most covered topics in drug delivery research. One potential drug carrier solution is the use of materials that are already commercially available in ophthalmic lenses for the correction of refractive errors. In this study, we present a diffusion-based mathematical model in which the parameters can be adjusted based on experimental results obtained under controlled conditions. The model allows for the design of multi-layered therapeutic ophthalmic lenses for controlled drug delivery. We show that the proper combination of materials with adequate drug diffusion coefficients, thicknesses and interfacial transport characteristics allows for the control of the delivery of drugs from multi-layered ophthalmic lenses, such that drug bursts can be minimized, and the release time can be maximized. As far as we know, this combination of a mathematical modelling approach with experimental validation of non-constant activity source lamellar structures, made of layers of different materials, accounting for the interface resistance to the drug diffusion, is a novel approach to the design of drug loaded multi-layered contact lenses.

## Introduction

The use of ophthalmic lenses (OLs) as drug carriers has been well established among the scientific community as a promising technique for increasing the efficacy of several ophthalmological therapies [1,2]. The main advantage of using OLs as drug carriers is that the immediate, uncontrolled delivery of drugs, such as by conventional eye drops and intravenous solutions, is prevented. Moreover, drugs instilled as eye drops have a short residence time in the tear film, leading to a low drug bioavailability [3,4]. Therapeutic OLs may increase the ocular residence time of drugs, minimizing drug waste and side effects [1]. However, to achieve these goals, therapeutic OLs must deliver the drug in a controlled manner. To do so, important questions that have impaired the commercial availability of such drug delivery systems must be solved. The main drawbacks that remain to be solved include the initial burst of drug release and the limited duration of active release after the placement of the lens in the eye. The simplest approach for preparing drug-loaded OLs, which has been the focus of many previous studies, involves soaking hydrophilic lenses in commercial drug solutions [2,5,6]. This minimalistic approach, although leading to more effective drug release control and drug absorption than eye drops, in general, cannot provide extended drug release [7,8]. To increase the temporal profile of drug release, several approaches have been suggested. Gulsen et al. [9] developed nanoparticle-laden gels capable of loading substantial amounts of drug that could be released at a controlled rate. Hiratani et al. [10] and Ali et al. [11] proposed ‘imprinted’ contact lenses, which led to a significant increase in partition coefficients and to slower drug release. Anderson et al. [12] modified the surface of poly(2-hydroxyethylmethacrylate) (P(HEMA)) gels with an n-alkyl coating to produce a hydrophobic rate-limiting barrier that controlled the release of antibiotics. More recently, our group showed that plasma treatment may reduce the initial drug release kinetics of drug-loaded contact lenses made of a P(HEMA)-based hydrogel and a silicone-based hydrogel [13]. Although the approaches listed above are effective in increasing the drug release duration, longer periods of release are still required, especially in the case of intraocular lenses, which are designed to remain inside the eye.

A promising approach for therapeutic OLs recently presented by Ciolino and co-workers [14,15] was the use of a sandwich structure that encapsulated a drug-containing film in a different drug-free polymeric matrix. The authors developed a so-called drug-eluting contact lens capable of delivering glaucoma medication to the eye in a sustained manner for at least four weeks [14,15]. Despite the extended temporal duration of the drug release, the authors referred to an unavoidable initial drug release burst, which they attempted to reduce by pre-conditioning the OLs in PBS for a few days. However, this pre-conditioning step led to a reduced steady-state concentration.

The mathematical modeling of the different mechanisms responsible for controlled release from hydrogels such as diffusion, swelling or degradable controlled systems is well described in literature [16,17]. Siepmann and Siepmann described analytically the mass transport from different diffusion controlled drug delivery systems including from a reservoir type slab [18]. Mathematical description and characterization of the release behavior allows the prediction and selection of the system parameters in order to tailor the drug release profile.

The aim of this work was to describe and characterize through a diffusion-based mathematical model the design of multi-layered drug-loaded lenses with optimal drug release behavior. The model of the drug release profiles of the devices is described, and the results are compared with experimental data. Then, we used the model to predict the drug release behavior of a specially manufactured multi-layered lens loaded with levofloxacin and chlorhexidine and to analyze the contributions of the parameters of multi-layered OLs, namely the roles of the drug diffusion coefficient in the material and the thickness and interfacial transport characteristics of the layers. We show that the initial burst may be minimized, and near zero-order release conditions may be achieved by properly selecting the relative dimensions and characteristics of the loaded/non-loaded layers of the lenses.

## Materials and Methods

2-hydroxyethyl methacrylate (HEMA), ethylene glycol dimethacrylate (EGDMA), 2,2-azobis(2-methylpropionitrile) (AIBN) and levofloxacin (LVF) were all purchased from Sigma-Aldrich. Poly(vinylpyrrolidone) (PVP, KollidonVR 30) was kindly provided by BASF. Sodium chloride was obtained from Merck, and chlorhexidine diacetate monohydrate (CHX) was obtained from AppliChem. A Millipore Milli-Q water purification system was used to prepare distilled and deionized (DD) water.

### Hydrogel preparation and drug loading and release experiments

A P(HEMA)-based hydrogel (HEMA/PVP) was prepared by dissolving appropriate amounts of the EGDMA crosslinker and the AIBN initiator in HEMA to obtain final concentrations of 80 mM and 10 mM, respectively. PVP was added to the mixture at a ratio of 98/2 HEMA/PVP (w/w). The mixture was poured into a mold that consisted of two parallel silanized glass plates, and the mixture was thermopolymerized at 50°C for 14 h followed by 24 h at 70°C. The obtained hydrogel sheet was soaked in DD water for 5 days to remove unreacted monomers, cut into discs (2 cm^2^, average thickness of 0.3 mm), which were then dried in an oven at 40°C overnight and stored. Additional details on the protocol followed for the preparation of the hydrogel can be found in Paradiso et al. [6].

Levofloxacin was dissolved in saline solution (130 mM) at concentrations of 5 mgmL^-1^ and 10 mgmL^-1^. Chlorhexidine was dissolved in DD water at concentrations of 1 mgmL^-1^ and 2.5 mgmL^-1^ due to its reduced solubility in saline solution. The hydrogels were drug loaded by soaking each disc in 5 mL of the drugs solutions for 5 days at 4°C. *In vitro* drug release tests were performed at 37°C while stirring (180 rpm) 5 mL saline solution until the release of the drug was complete. At chosen time intervals, aliquots of 0.5 mL of the supernatant were collected and replaced by the same volume of fresh saline solution. The drug concentration values in the release medium were quantified using a spectrophotometer UV–VIS MultiscanGO from Thermo Scientific^®^ at wavelengths of 255 nm for CHX and 290 nm for LVF. All the experiments were carried out in triplicate.

**Fig 1:**
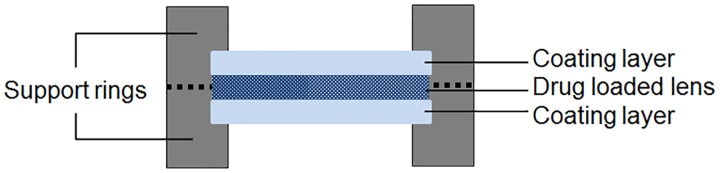
Schematic representation of the experimental multi-layered drug-releasing system.

To experimentally simulate the multi-layered lens system, a support ring made of *Perspex*^®^ acrylic was designed. A levofloxacin-loaded HEMA/PVP disc (loaded with a 5 mgmL^-1^ solution) was placed between two non-loaded HEMA/PVP discs inside the support, and the disks were pressed against one another in the peripheral zone. A schematic representation of the experimental multi-layered system is presented in Fig 1. *In vitro* drug release was characterized in a volume of saline solution proportional to the volume used in the single-lens drug release assays, i.e., maintain the ratio of the cross-sectional area/volume of the supernatant.

### Mathematical model for simulation of in vitro drug release—monolayered lens

**Fig 2:**
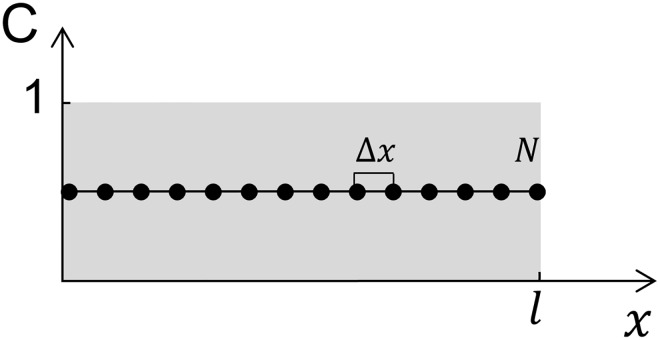
Schematics of the implementation of the diffusion model for a homogeneous lens initially loaded with a homogeneous normalized unitary concentration of drug $C_{k}^{t=0}$, where k = [1, N − 2].

The simplest case was used to describe the drug release from the drug-loaded homogeneous lens immersed in a liquid wherein the lens was considered a plate of infinite surface area and finite thickness, *l*, and Fick’s second law of diffusion was applied, which is an approach similar to that recently used by Pascoal, Silva and Pinheiro [16]. Assuming drug diffusivity, D, independent of time and space and taking the space coordinate x orthogonal to the surface of the lens with the origin at the left edge of the lens (Fig 2), the mass transfer problem considering a material with a certain concentration of drug (*C*) can be described using the following equation:

$$\frac{dC}{dt}=D\left(\frac{\partial^{2}C}{dx^{2}}\right)$$ (1)

The initial and boundary conditions for a lens immersed in a drug-free medium (*C*_*Ext*_) are as follows:

$$C\left(x,0\right)=C_{o},\;x\in \left[0,l\right]$$ (2.1)

$$\frac{dC}{dx}\left(\frac{l}{2},t\right)=0,\;t\geq 0$$ (2.2)

$$C_{Ext}=0,\;t\geq 0$$ (2.3)

$$-D\frac{\partial C}{\partial x}\left(0,t\right)=\;\alpha \;\left(C\left(0,t\right)-\;C_{Ext}\right),\;t>0\text{ and }D\frac{\partial C}{\partial x}\left(l,t\right)=\alpha \;\left(C\left(l,t\right)-\;C_{Ext}\right),\;t>0$$ (2.4)

The initial condition 2.1 states that the lens at *t* = 0 has a uniform concentration of drug, *C*_*o*_. Conditions 2.2 and 2.3 define, first, a standard symmetry condition where $\frac{l}{2}$ is half of the thickness of the lens and, second, that the concentration of the drug at the exterior of the lens, *C*_*Ext*_, is always null. Finally, Eq 2.4 represent the boundary conditions where *α* accounts for the resistance to mass transport, in this case considered null (*α* = 1).

As shown in Fig 2, *N* represents the number of spatial nodes of the system, and Δ*x*, the distance between nodes, is given by $\Delta x=\frac{l}{N}$. Respecting the initial condition stated in Eq 2.1, at *t* = 0, the normalized initial value of the concentration is $C_{k}^{t=0}=1,\;k=\left[1,N-2\right]$ because we considered that, at the boundary nodes (1 and *N*), the drug concentration is equal to the concentration in the exterior medium. The time step was fixed at 1 minute and the Crank-Nicolson implicit method was used to solve Eq 1.

**Fig 3:**
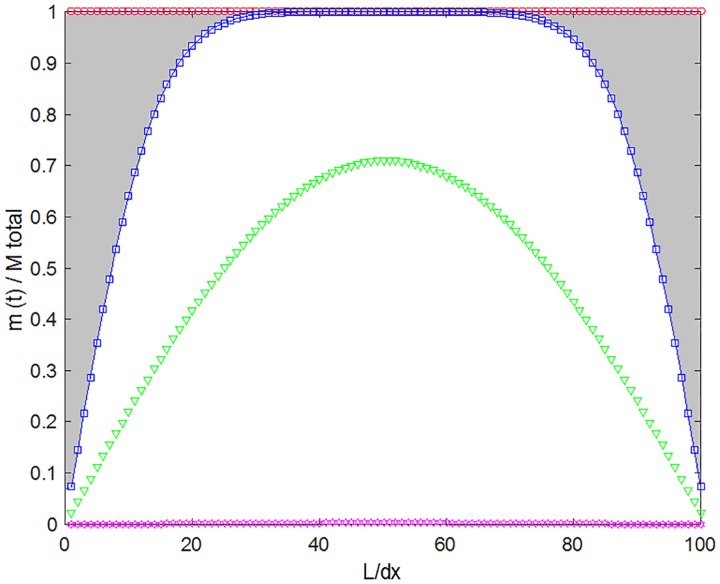
Evolution with time of the fractional drug mass in the lens. Time (minute) 0 (circles), 100 (squares), 1000 (triangles) and 10000 (stars); the profiles were obtained for a lens with a thickness of 1 mm and a diffusivity of 1x10^-12^ m^2^s^-1^.

Fig 3 shows the evolution of $C_{k}^{t}$ with time, where the inner part of the lens is maintained at a higher concentration than the outer borders during all of the release period, as expected. At each time, the (normalized) amount of drug released is given by the difference between the initial amount of drug and the total amount of drug that remains in the lens. For example, the normalized released amount for *t = 100 min* is represented by the shadowed area in Fig 3 (delimited by the line and squares). This approach allows the numerical simulation results to be directly compared with the experimental results.

### Mathematical model for simulating in vitro drug release—multi-layered lenses (drug-loaded core with a non-loaded coating)

**Fig 4:**
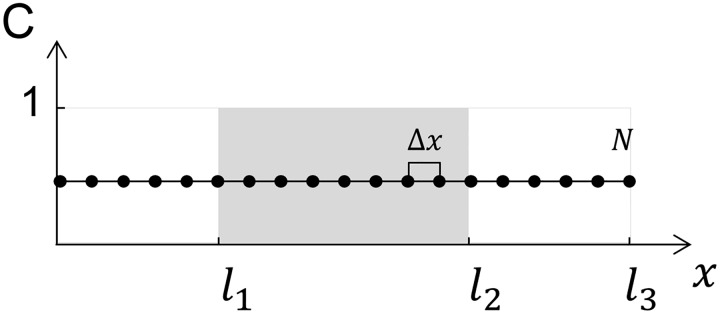
Schematics of the implementation of the diffusion model for a homogeneous drug-loaded lens initially loaded with a homogeneous normalized unitary concentration, $C_{k}^{t=0}$, and coated on both sides with a non-loaded diffusion barrier.

In this section, we assume the ophthalmic lens as a composite sandwich in which each layer is characterized by a certain thickness and a certain diffusivity (*D*) of the drug loaded within it. Only the inner layer is loaded with the drug, as schematically shown in Fig 4.

For this system, the Crank-Nicolson scheme was also used. In this case, the drug diffusivity in the coated layer and in the inner-loaded layer may be equal or different. The thicknesses of the lateral coatings ([0, *l*_1_] and [*l*_2_, *l*_3_]) are equal. This system can be described by *C*_*in*_ and *D*_*in*_ as the drug concentration and diffusivity, respectively, of the interior layer and by *C*_*out*_ and *D*_*out*_ as the drug concentration and diffusivity, respectively, of the outer layers, with the following conditions:

$$C_{in}\left(x,0\right)=C_{o},\;x\in \left[l_{1},l_{2}\right]$$ (3.1)

$$C_{out}\left(x,0\right)=0,\;x\in [0,\;l_{1}[\;or\;x\in ]l_{2},l_{3}]$$ (3.2)

$$\frac{dC_{in}}{dx}\left(\frac{l_{total}}{2},t\right)=0,\;t\geq 0$$ (3.3)

$$C_{Ext}=0,\;t\geq 0$$ (3.4)

$$D_{in}\frac{\partial C_{in}}{\partial x}\left(\;l_{1},t\right)=D_{out}\frac{\partial C_{out}}{\partial x}\left(\;l_{1},t\right),\;t>0\text{ and }D_{in}\frac{\partial C_{in}}{\partial x}\left(\;l_{2},t\right)=D_{out}\frac{\partial C_{out}}{\partial x}\left(\;l_{2},t\right),\;t>0$$ (3.5)

$$-D_{in}\frac{\partial C_{in}}{\partial x}\left(\;l_{1},t\right)=\alpha \;\left(C_{in}\left(\;l_{1},t\right)-\;C_{out}\left(\;l_{1},t\right)\right),\;t>0\text{ and }D_{in}\frac{\partial C_{in}}{\partial x}\left(\;l_{2},t\right)=\alpha \;\left(C_{in}\left(\;l_{2},t\right)-\;C_{out}\left(\;l_{2},t\right)\right),\;t>0$$ (3.6)

At *t* = 0 only the inner layer has a certain concentration of drug, *C*_*o*_, with the exterior layers having null drug concentrations. As in the system described in the previous section, a symmetry condition is imposed at half of the thickness of the total system length ($\frac{l_{total}}{2}$), and the concentration in the exterior medium is maintained null. Resistance to the mass transport through the interfaces between the interior and exterior layers is accounted for with an adjustable α.

## Results and Discussion

### Adjustment to experimental results: Determination of system parameters

The initial condition for the lens, either for the single-layer model Eq (2.1) or the multi-layer model Eq (3.1) requires that at the beginning of the experiment, the concentration of drug through the entire lens must be constant. Therefore, soaking in the drug solution should be sustained for a sufficient amount of time to ensure drug homogeneity in the lens. Different loading times were tested, and it was concluded that 5 days was enough time to achieve this condition. To ensure that the concentration of drug in the surface of the lens is zero at t > 0, the release was performed while stirring and within a sufficiently large volume of medium, which could be considered as infinitely diluted (sink conditions).

To compare the experimental profiles with the calculated profiles, the released mass was normalized, i.e., the mass released up to time t, M(t), was divided by the total mass M(∞). To solve the model system, a normalized initial value of $C_{k}^{t=0},\;k=\left[1,N-2\right]$ was considered, and a constant volume of the lens was assumed. Then, the numerically calculated normalized profiles can be directly compared with the normalized experimental profiles, and the adjustable parameters D and α (α for multi-layered systems) can be extracted.

**Fig 5:**
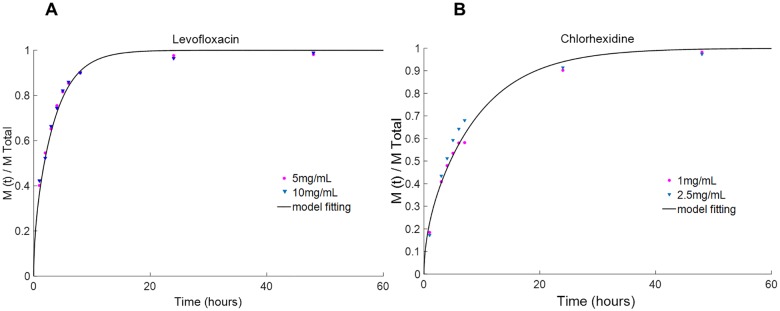
Adjustment of the numerically fit models to experimental points obtained from the release assays for infinite sink conditions. (A) levofloxacin and (B) chlorhexidine from HEMA/PVP hydrogels. The concentrations are given in the inserts.

The first step was to adjust our model to the experimental results derived from the non-coated lenses to obtain the diffusivity values of the studied drugs in the studied material. Fig 5 shows two examples of theoretical curves fitted to experimental points for two different lens/drug systems: a HEMA/PVP-levofloxacin system (Fig 5a) and a HEMA/PVP-chlorhexidine system (Fig 5b).

For each drug, two different concentrations of soaking solution were used. The lenses that were soaked in more concentrated solutions had released higher amounts of drug by t = ∞. However, the normalized experimental curves for the two soaking conditions did not present significantly different release kinetics for the studied drugs, as was expected because the diffusivity of the drug is independent of its concentration.

This finding is illustrated in Fig 5, where the experimental data points refer to the normalized mass release of levofloxacin and chlorhexidine loaded from solutions of different concentrations. As a result, it can be concluded that the diffusivity values are independent of the concentration of the soaking solution.

The diffusivity values of the different studied systems determined by our model were compared with those derived based on an analytical solution to Fick’s second law of diffusion, as described by Peng et al. [19]. An agreement between the two sets of values was obtained (Table 1).

**Table 1:** Drug diffusivity (D) of chlorhexidine and levofloxacin in HEMA/PVP hydrogels.

	D (m^2^s^-1^)	D [Peng et al. [19]] (m^2^s^-1^)
Chlorhexidine	5.0x10^-13^	4.0x10^-13^
Levofloxacin	7.5x10^-13^	6.7x10^-13^

The second step in adjusting the mathematical model to experimental results was to obtain an estimate for α, the parameter related to the mass transfer within the interface between the loaded and non-loaded lens in the multi-layered system. Experimental data were obtained for a levofloxacin-loaded HEMA/PVP lens compressed between two non-loaded HEMA/PVP lenses with the same thickness as the loaded lens (0.4 mm/layer). The diffusivity of 7.5x10^-13^ m^2^s^-1^ corresponded to the value previously obtained for this drug in the HEMA/PVP system.

**Fig 6:**
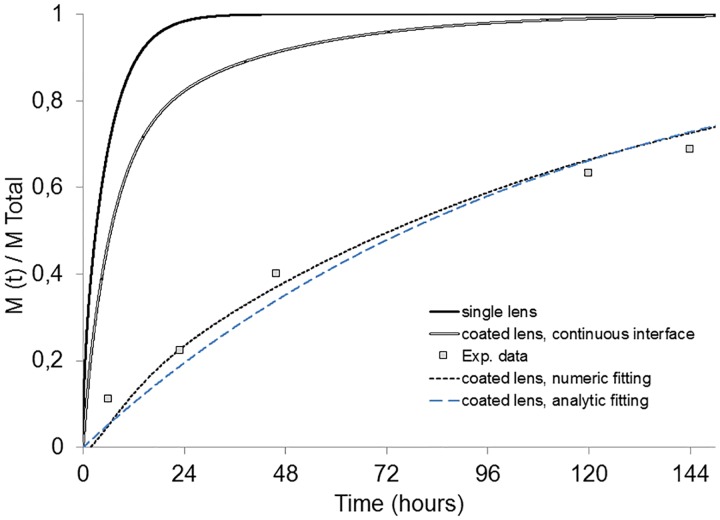
Predicted fractional release mass profiles given by numerical simulation. Single HEMA/PVP lens (“single inner”) and coated HEMA/PVP lens with α = 1 (“coated lens, continuous interface”); comparison of experimental results of levofloxacin release from a HEMA/PVP multi-layered system (“Exp. data”) fitting through a numeric solution (“coated lens, numeric fitting”) and a analytic solution (Equation (3) in Reference [18]) (“coated lens, analytic fitting”) of Fick’s second law of diffusion.

The adjustment to the experimental release data, as shown in Fig 6 (doted gray curve), revealed that an *α* = 0.07 resulted in an almost perfect adjustment between the experimental points and the model trend line, indicating that the interfacial resistance to mass transport in the multi-layered OLs systems is a key factor in drug release control. In fact, in this example, the introduction of the discontinuity across the interface between the loaded and non-loaded layers showed that the multi-layered system resulted in an increase in the total release time from 20 hours to more than 150 hours.

Fitting of the experimental data to a general analytic solution of Fick’s law described by Siepmann and Siepmann (Equation (3) in Reference [18]) is also presented in Fig 6 (dashed blue curve). The same diffusivity was used and the fitting was optimized by adjusting the partition coefficient, k. An optimized k = 0.043 (which somehow compares with the α of the numerical model) was obtained. Though both fits are acceptably good (although slightly worst with the analytical model) it should be noted that:

1. the analytic solution, for the sake of simplicity, only accounts for the diffusivity through the external layer;
2. conversely, the numerical solution accounts for both the external and inner layer diffusivities which can be dissimilar and determined a priori for the design of optimized systems (treated in the final section of the present paper);
3. finally, the analytic model does not predict the delay time of the drug crossing the outer layers, which, in a more realistic manner, is predicted by the numerical model.

### Application of the design of the multi-layered drug-loaded lens

In this section, we utilize the previously presented approach in two example applications. In the first example, we establish as target objective multi-layered HEMA/PVP lenses that could release levofloxacin and chlorhexidine over the course of one week at a nearly zero-order release rate. To simulate these model lenses, we used the diffusional and interfacial parameters determined in the previous section. Then, we manufactured the lenses (as described in section *Hydrogel preparation and drug loading and release experiments*) and compared the predicted release results with the experimental release results. In the second example application of the model, we used the approach in a general manner to analyze the influence of the drug/lens parameters of the obtained release profiles.

### Slow-release multi-layered HEMA/PVP lens loaded with levofloxacin and chlorhexidine: Simulation and experimental results of the model systems

As shown in the previous section, by using the calibrated drug diffusivity parameters and after gauging the α interface parameter, it is possible to numerically simulate different systems to obtain optimal multi-layered lenses for desired applications. Preliminary calculations showed that, for the HEMA/PVP system and for a typical lens thickness of 1.2–1.6 mm, if the loaded core is approximately the same thickness as the un-coated layers, a slow drug release that can release the drug at nearly a constant release rate with minimal drug bursts over the course of a week can be achieved. The first system (#1) that we modeled and tested consisted of a lens formed by a drug-loaded core of 400 μm and coated un-loaded layers of the same size (400 μm), such that the total thickness of the model lens was 1.2 mm. A α factor of 0.07 was obtained previously from fitting system #1 experimental data to the numerical model and was used for the following simulation. The second system (#2) consisted of a drug-loaded core of 400 μm and coated un-loaded layers of 600 μm, such that the total thickness of the model lens was 1.6 mm. For both model systems, we used levofloxacin (diffusivity of 7.5x10^-13^ m^2^s^-1^) and chlorhexidine (diffusivity of 5x10^-13^ m^2^s^-1^) as the release drugs in the experimental validation.

**Fig 7:**
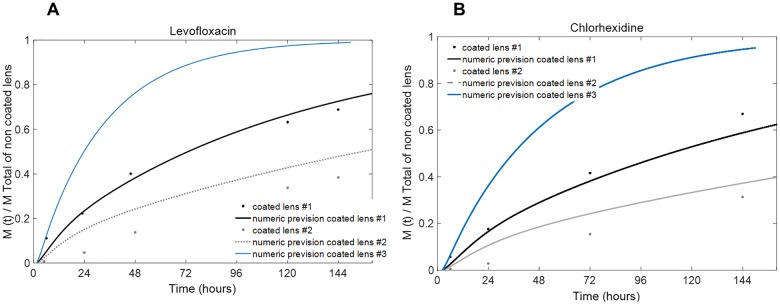
Predicted fractional release mass profiles given by the numerical simulation for coated HEMA/PVP lens systems. Inner and outer lens thicknesses of 0.4 mm/layer (coated lens #1), inner lens thickness of 0.4 mm and an outer lens thickness of 0.6 mm/lens (coated lens #2), and inner lens thickness of 0.4 mm and an outer lens thickness of 0.2 mm/lens (coated lens #3). Experimental release experiments data (black and gray dots) for (A) levofloxacin and (B) chlorhexidine.

Fig 7 shows a numerical simulation of the drug release from these systems (the first and second systems are designated as #1 and #2, respectively, in the figure) compared with the experimental points measured after 6, 24, 48, 72, 120 and 144 h of release. For system #2, numerical predictions for both levofloxacin and chlorhexidine slightly overestimate the release profiles when compared to the experimentally obtained curves. We observed that the increase in the coating thickness by a factor of 1.5 significantly affected the absolute value of the drug release with time but not the release kinetics. In fact, for both of these model lenses and for both drugs tested, we observed that, theoretically and experimentally, after the first day, essentially a zero-order release rate was obtained up to at least 150 h of release. Predictions for coatings with half of the thickness of system #1 are also presented and are designated as system #3.

The results presented in this section support the hypothesis that the experimental release profile of a coated lens can be tailored by the parameters of the overall system. In the next section, we assume this premise to provide a general overview of the influence of the control parameters (diffusion coefficient, interfacial transfer coefficient and thickness of the lens) on the drug release profiles.

### Design of multi-layered drug-loaded lens: A generalist approach

Next, we present illustrative cases for which the thickness of the coating, the diffusivity of the drug in the coating, and the interface mass transport resistance parameter are varied. In addition to the predicted fractional mass release profiles, the predicted normalized drug concentration profiles if the systems were placed, as intraocular lenses, in the eye aqueous humor (volume of 0.250 mL) and assuming a physiological renovation rate of 1% per minute were also considered [20]. The predicted drug concentrations were estimated from the theoretical fractional mass release profiles based on a mathematical model described in Paradiso el al. [5]. Here, the thickness of the inner loaded lens was maintained at 0.5 mm, and the drug diffusivity in that material was maintained at 7.5x10^-13^ m^2^s^-1^.

**Fig 8:**
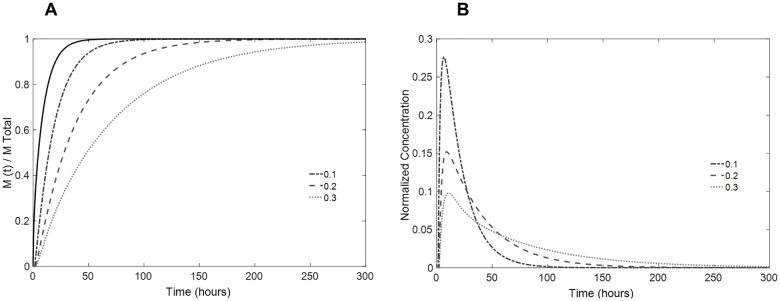
Influence of the coating thickness in the drug release. (A) predicted fractional release mass profiles given by numerical simulation; (B) estimated normalized concentration of drug in the aqueous humor volume taking into account its renovation rate for coated lenses. Coating thickness values (in mm/coating layer) are shown in the figure (full black line: single lens; dashed lines: coated lenses).

Fig 8 shows the influence of the coating thickness on the release profile of the lens, keeping the drug diffusivity (D_inner lens_ = D_coating_ = 7.5x10^-13^ m^2^s^-1^) and the mass transfer related parameter (*α* = 0.07) constant. It can be observed that by increasing the thickness of the coating, the total time to release the drug increases because the drug must traverse a greater distance. More interesting is the decrease in the initial burst of drug (Fig 8b) with increased coating thicknesses.

**Fig 9:**
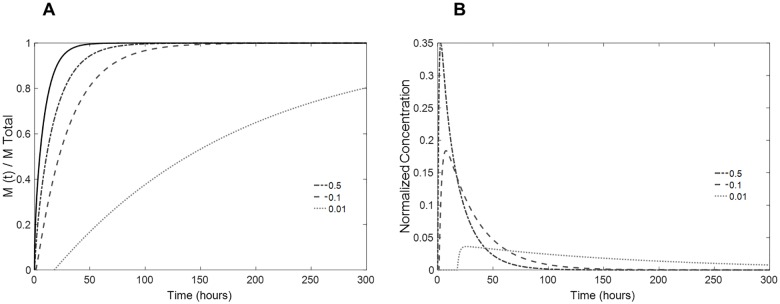
Influence of the resistance to the mass transport through the interfaces in the drug release. (A) predicted fractional release mass profiles given by numerical simulation; (B) estimated normalized concentration of drug in the aqueous humor volume taking into account its renovation rate in coated lenses. The values of α are shown in the figure (full black line: single lens; dashed lines: coated lenses).

Next, the effect of resistance to the mass transfer at the coating interface was estimated by altering the parameter α. Drug diffusivity in the coating was maintained equal to the diffusivity in the inner lens (D_inner lens_ = D_coating_ = 7.5x10^-13^ m^2^s^-1^). The coating thickness was set at a fixed value of 0.2 mm on each side. Fig 9 shows the dependence of the resulting mass release profile and concentration burst on this adjustable parameter.

Note that by decreasing the parameter α by one order of magnitude, a significant change occurs in the release kinetics. With *α* = 0.01, an almost zero-order release is achieved. In addition, the time lag for drug release increases (abscissa axis; Fig 9a) due to the resistance to drug transport in the interface. This lag time must be accounted for very carefully in drug delivery ophthalmic lenses because, during this time period, no drug would be available in the eye. The initial drug burst can be significantly decreased if the mass transfer in the interface is precisely calibrated.

**Fig 10:**
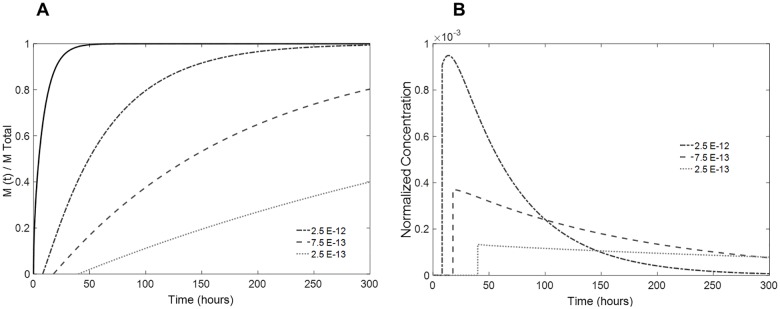
Influence of the coating drug diffusivity in the drug release. (A) predicted fractional release mass profiles given by numerical simulation; (B) estimated normalized concentration of drug in the aqueous humor volume taking into account the renovation rate of coated lenses. Coating diffusivity values are shown in the figure (full black line: single lens; dashed lines: coated lenses).

The role of the drug diffusivity in the coating was also assessed by maintaining its thickness at a fixed value of 0.2 mm on each side and considering *α* = 0.01. As shown in Fig 10, by decreasing the drug diffusivity in the coating to one-third of the diffusivity in the drug-loaded lens, the kinetics of the mass release are greatly altered, and the mass is released at lower rate. Note that the burst and the time lag are also markedly affected by this variation. In contrast, if the drug diffusivity of the coating is superior to that of the lens, the total mass release occurs more quickly.

Considering the above results and the requirements for efficient drug release, a theoretical optimized multi-layered intraocular lens can be designed based on the input values given in Table 2.

**Table 2:** Input values for numeric simulation.

*Lens thickness (mm)*	0.5
*Coating thickness (mm)*	0.2
α	0.01
*Drug diffusivity in the lens (m*^*2*^*s*^*-1*^*)*	7.5x10^-13^
*Drug diffusivity in the coating (m*^*2*^*s*^*-1*^*)*	7.5x10^-13^

The fractional mass release profile and expected normalized drug concentration in the aqueous humor for use of this theoretically designed lens are depicted in Fig 10. A theoretical optimal ophthalmic lens depends on multiple factors (physiological, pharmacokinetics, etc.) and on the desired application (treatment requirements). Here, we aimed to achieve an effective intraocular lens that could be used during the critical period after cataract removal surgery to prevent the development of postoperative endophthalmitis.

The initial time lag of this multi-layered system was estimated to be approximately 24 hours, corresponding to the time period during which antibiotic intracameral injections that are commonly applied following this type of surgery are estimated to be effective [21]. After this time lag, the release of drug from the multi-layered system is sustained for a period of at least 12 days and is likely sufficient to prevent acute endophthalmitis, which most likely develops within 1–2 weeks after surgery [22].

It must be taken into account that the concentration values presented in Figs 8–10 are estimated from a fractional release mass curve and are, therefore, normalized. As mentioned above, the total mass released and, consequently, the *in vivo* drug concentration are dependent on the drug-soaking solution concentration, which determines the total mass of drug uptake. A lens loaded with a solution of a higher drug concentration will release greater amounts of mass while not affecting the kinetics of release, as demonstrated in Fig 5.

## Conclusions

As previously described, the primary aim of ocular drug release studies is to minimize the initial burst of drug release and to achieve a constant target release rate over an adequate time interval. Coating the drug-loaded lenses is a common strategy adopted to achieve these aims. Here, a mathematical model based on a numerical solution of Fick’s second law of diffusion is proposed to predict how a certain coating influences the drug release profile from a given material. The model predictions were compared with experimentally obtained results to validate the model and were then used to predict the behavior of the drug-loaded multi-layered lens. This work shows that by properly controlling the materials of a multi-layered lens and the interfacial mass flux properties, controlled drug delivery can be achieved. Additionally, by manipulation of the system characteristics (e.g., thickness of the layers, diffusivity of the drugs), a tailored drug release profile can be designed to achieve the desired therapy.

## Supporting Information

S1 Data Experimental results obtained from drug release experiments. (XLSX)
